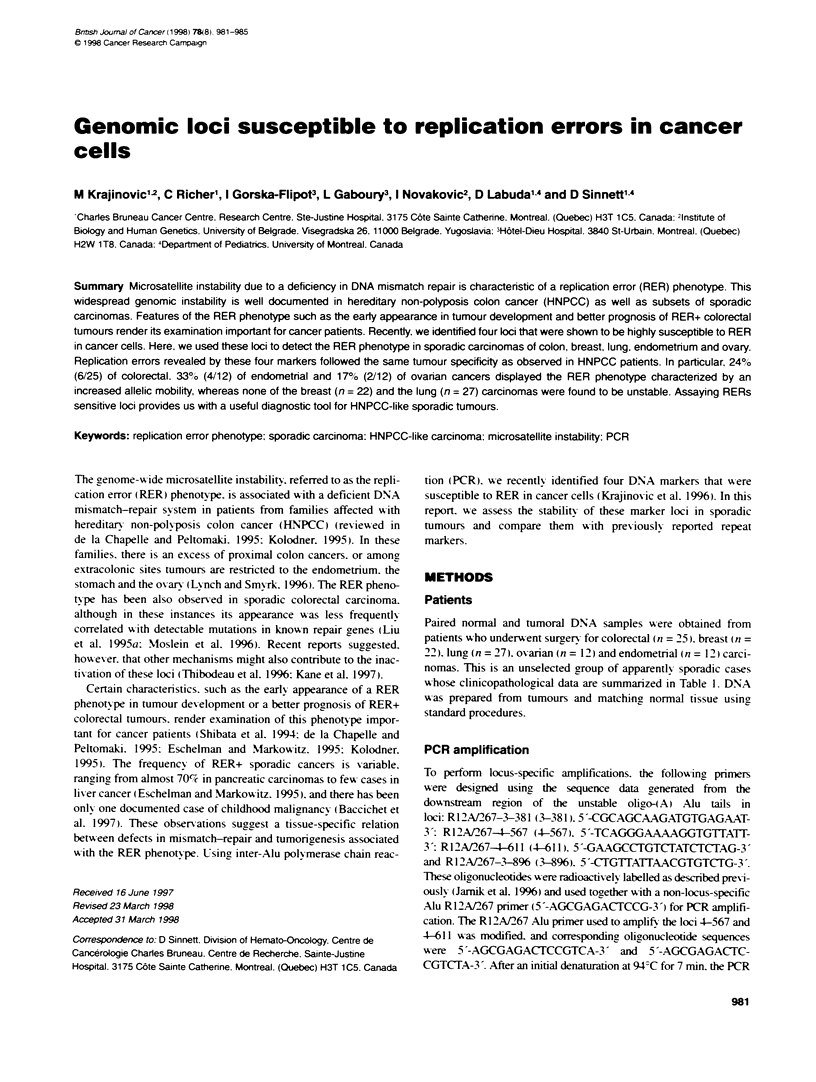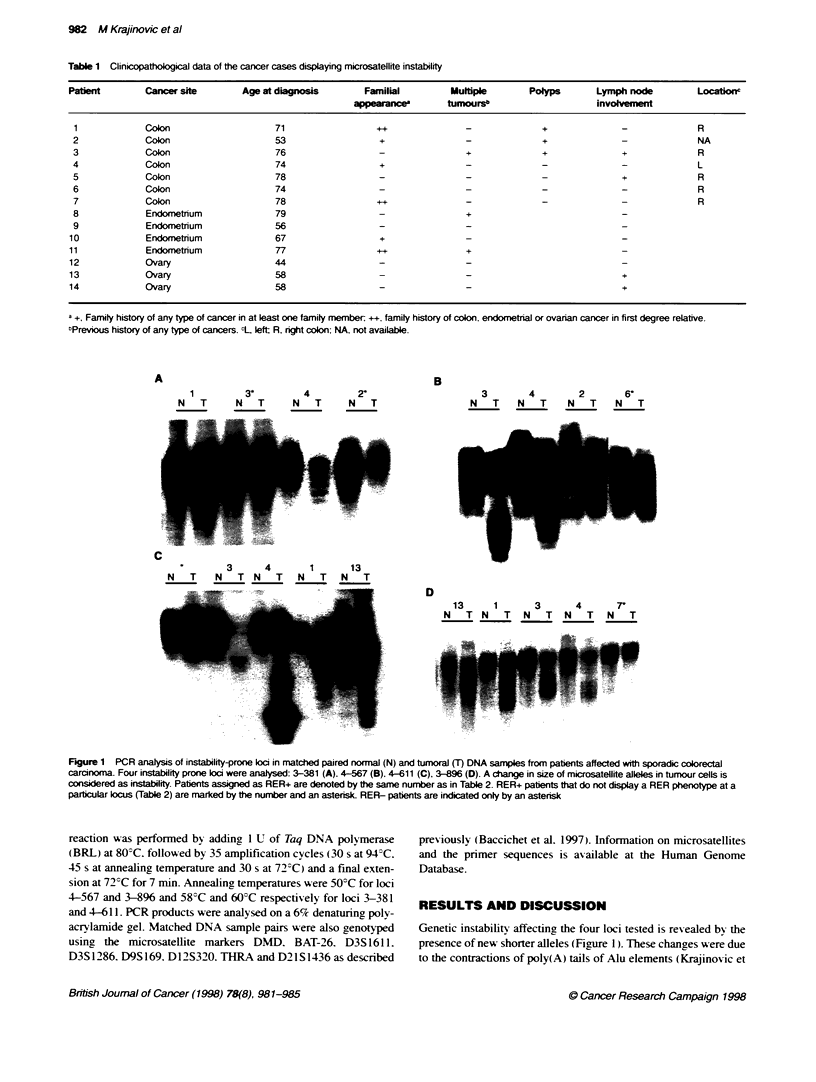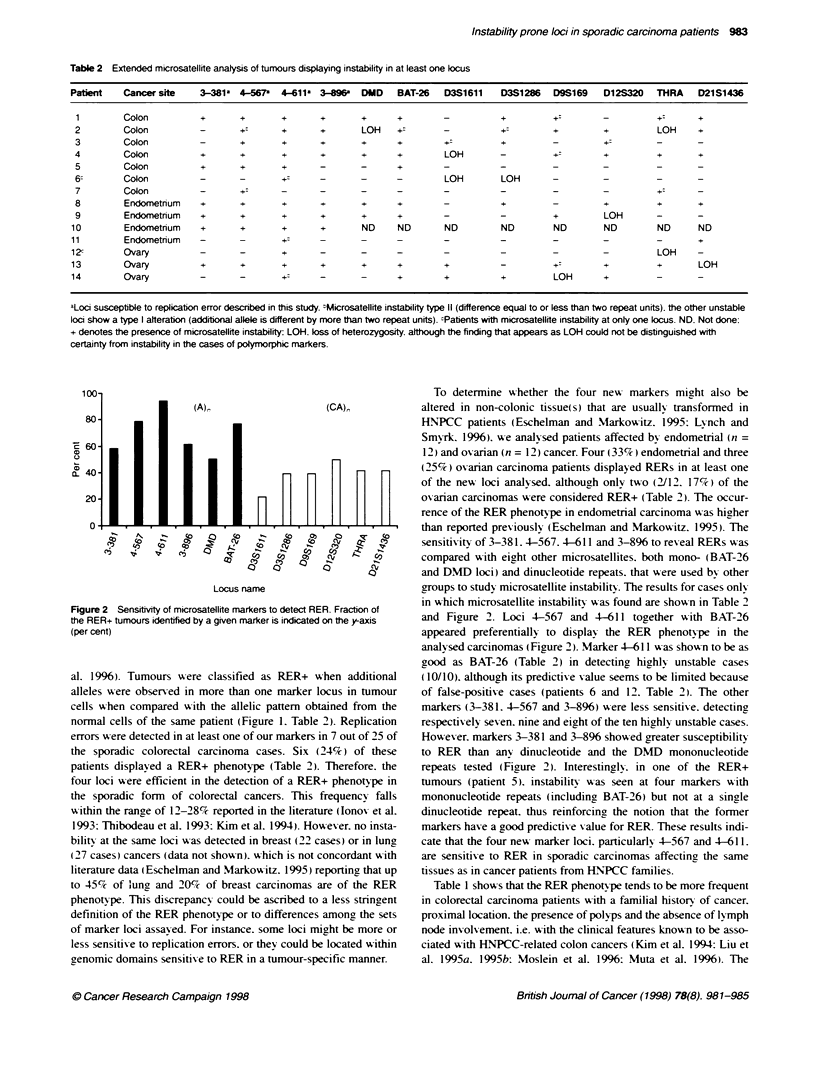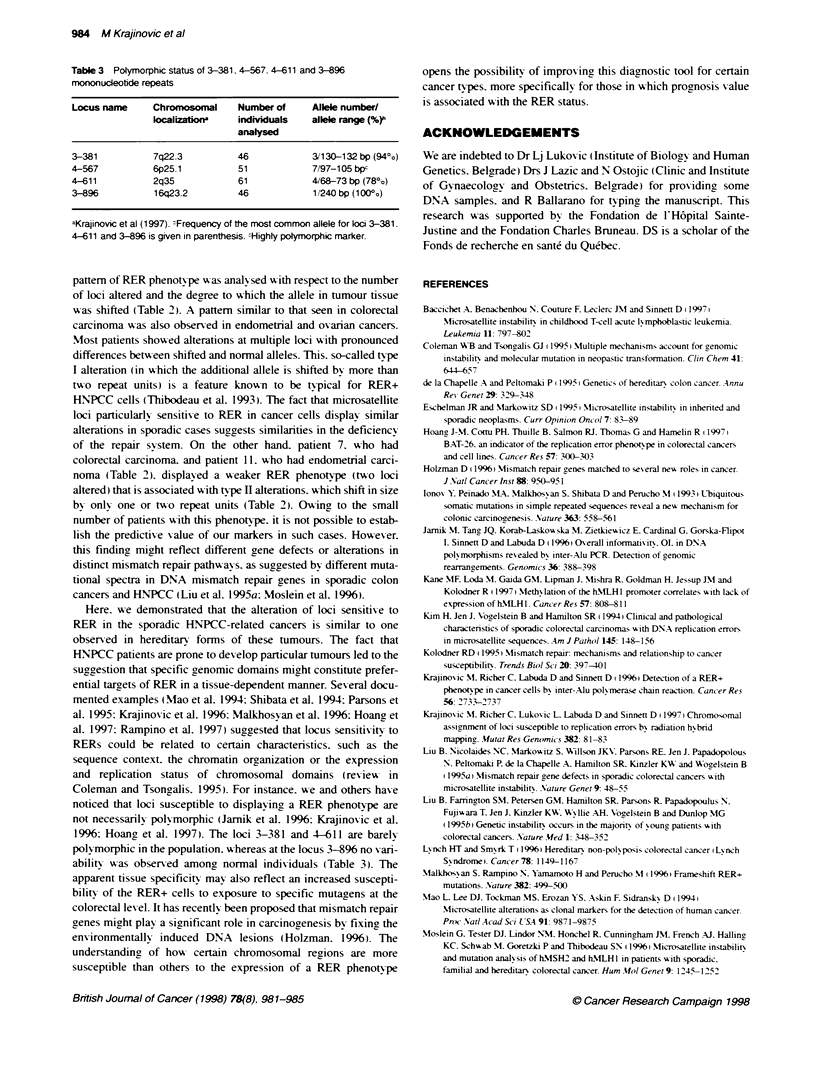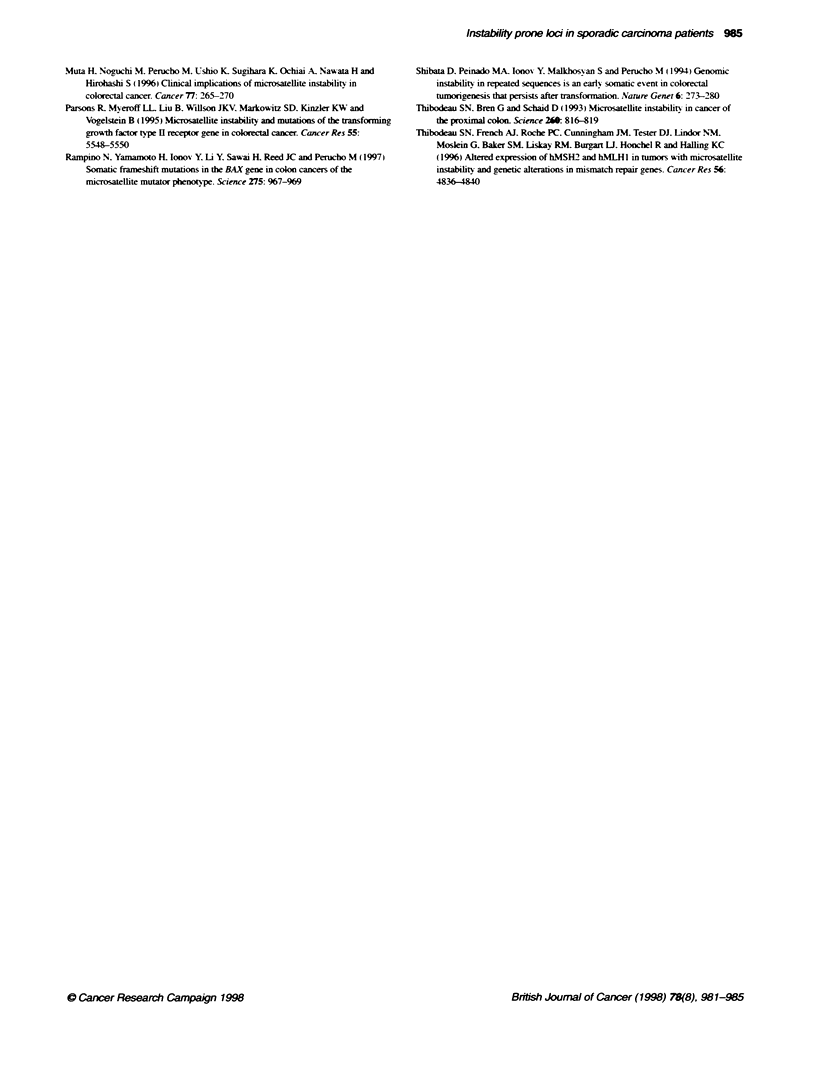# Genomic loci susceptible to replication errors in cancer cells.

**DOI:** 10.1038/bjc.1998.616

**Published:** 1998-10

**Authors:** M. Krajinovic, C. Richer, I. Gorska-Flipot, L. Gaboury, I. Novakovic, D. Labuda, D. Sinnett

**Affiliations:** Charles Bruneau Cancer Centre, Research Centre, Ste-Justine Hospital, Montreal, Quebec, Canada.

## Abstract

**Images:**


British Journal of Cancer (1998) 78(8), 981–985
© 1998 Cancer Research Campaign

### Genomic loci susceptible to replication errors in cancer cells

**M Krajinovic[1,2], C Richer[1], I Gorska-Flipot[3], L Gaboury[3], I Novakovic[2], D Labuda[1,4] and D Sinnett[1,4]**

[1]Charles Bruneau Cancer Centre, Research Centre, Ste-Justine Hospital, 3175 Côte Sainte Catherine, Montreal, (Quebec) H3T 1C5, Canada; [2]Institute of Biology and Human Genetics, University of Belgrade, Visegradska 26, 11000 Belgrade, Yugoslavia; [3]Hôtel-Dieu Hospital, 3840 St-Urbain, Montreal, (Quebec) H2W 1T8, Canada; [4]Department of Pediatrics, University of Montreal, Canada

**Summary** Microsatellite instability due to a deficiency in DNA mismatch repair is characteristic of a replication error (RER) phenotype. This widespread genomic instability is well documented in hereditary non-polyposis colon cancer (HNPCC) as well as subsets of sporadic carcinomas. Features of the RER phenotype such as the early appearance in tumour development and better prognosis of RER+ colorectal tumours render its examination important for cancer patients. Recently, we identified four loci that were shown to be highly susceptible to RER in cancer cells. Here, we used these loci to detect the RER phenotype in sporadic carcinomas of colon, breast, lung, endometrium and ovary. Replication errors revealed by these four markers followed the same tumour specificity as observed in HNPCC patients. In particular, 24% (6/25) of colorectal, 33% (4/12) of endometrial and 17% (2/12) of ovarian cancers displayed the RER phenotype characterized by an increased allelic mobility, whereas none of the breast ($n$ = 22) and the lung ($n$ = 27) carcinomas were found to be unstable. Assaying RERs sensitive loci provides us with a useful diagnostic tool for HNPCC-like sporadic tumours.

**Keywords:** replication error phenotype; sporadic carcinoma; HNPCC-like carcinoma; microsatellite instability; PCR

The genome-wide microsatellite instability, referred to as the replication error (RER) phenotype, is associated with a deficient DNA mismatch–repair system in patients from families affected with hereditary non-polyposis colon cancer (HNPCC) (reviewed in de la Chapelle and Peltomaki, 1995; Kolodner, 1995). In these families, there is an excess of proximal colon cancers, or among extracolonic sites tumours are restricted to the endometrium, the stomach and the ovary (Lynch and Smyrk, 1996). The RER phenotype has been also observed in sporadic colorectal carcinoma, although in these instances its appearance was less frequently correlated with detectable mutations in known repair genes (Liu et al, 1995*a*; Moslein et al, 1996). Recent reports suggested, however, that other mechanisms might also contribute to the inactivation of these loci (Thibodeau et al, 1996; Kane et al, 1997).

Certain characteristics, such as the early appearance of a RER phenotype in tumour development or a better prognosis of RER+ colorectal tumours, render examination of this phenotype important for cancer patients (Shibata et al, 1994; de la Chapelle and Peltomaki, 1995; Eschelman and Markowitz, 1995; Kolodner, 1995). The frequency of RER+ sporadic cancers is variable, ranging from almost 70% in pancreatic carcinomas to few cases in liver cancer (Eschelman and Markowitz, 1995), and there has been only one documented case of childhood malignancy (Bacchichet et al, 1997). These observations suggest a tissue-specific relation between defects in mismatch–repair and tumorigenesis associated with the RER phenotype. Using inter-Alu polymerase chain reaction (PCR), we recently identified four DNA markers that were susceptible to RER in cancer cells (Krajinovic et al, 1996). In this report, we assess the stability of these marker loci in sporadic tumours and compare them with previously reported repeat markers.

*Received 16 June 1997*
*Revised 23 March 1998*
*Accepted 31 March 1998*

*Correspondence to:* D Sinnett, Division of Hemato-Oncology, Centre de Cancérologie Charles Bruneau, Centre de Recherche, Sainte-Justine Hospital, 3175 Côte Sainte Catherine, Montreal, (Quebec) H3T 1C5, Canada

### METHODS

### Patients

Paired normal and tumoral DNA samples were obtained from patients who underwent surgery for colorectal ($n$ = 25), breast ($n$ = 22), lung ($n$ = 27), ovarian ($n$ = 12) and endometrial ($n$ = 12) carcinomas. This is an unselected group of apparently sporadic cases whose clinicopathological data are summarized in Table 1. DNA was prepared from tumours and matching normal tissue using standard procedures.

### PCR amplification

To perform locus-specific amplifications, the following primers were designed using the sequence data generated from the downstream region of the unstable oligo-(A) Alu tails in loci: R12A/267–3–381 (3–381), 5′-CGCAGCAAGATGTGAGAAT-3′; R12A/267–4–567 (4–567), 5′-TCAGGGAAAAGGTGTTATT-3′; R12A/267–4–611 (4–611), 5′-GAAGCCTGTCTATCTCTAG-3′ and R12A/267–3–896 (3–896), 5′-CTGTTATTAACGTGTCTG-3′. These oligonucleotides were radioactively labelled as described previously (Jarnik et al, 1996) and used together with a non-locus-specific Alu R12A/267 primer (5′-AGCGAGACTCCG-3′) for PCR amplification. The R12A/267 Alu primer used to amplify the loci 4–567 and 4–611 was modified, and corresponding oligonucleotide sequences were 5′-AGCGAGACTCCGTCA-3′ and 5′-AGCGAGACTCCGTCTA-3′. After an initial denaturation at 94°C for 7 min, the PCR

**Table 1** Clinicopathological data of the cancer cases displaying microsatellite instability

| Patient | Cancer site | Age at diagnosis | Familial appearance[a] | Multiple tumours[b] | Polyps | Lymph node involvement | Location[c] |
|---|---|---|---|---|---|---|---|
| 1 | Colon | 71 | ++ | − | + | − | R |
| 2 | Colon | 53 | + | − | + | − | NA |
| 3 | Colon | 76 | − | + | + | + | R |
| 4 | Colon | 74 | + | − | − | − | L |
| 5 | Colon | 78 | − | − | − | + | R |
| 6 | Colon | 74 | − | − | − | − | R |
| 7 | Colon | 78 | ++ | − | − | − | R |
| 8 | Endometrium | 79 | − | + | | − | |
| 9 | Endometrium | 56 | − | − | | − | |
| 10 | Endometrium | 67 | + | − | | − | |
| 11 | Endometrium | 77 | ++ | + | | − | |
| 12 | Ovary | 44 | − | − | | − | |
| 13 | Ovary | 58 | − | − | | + | |
| 14 | Ovary | 58 | − | − | | + | |

[a] +, Family history of any type of cancer in at least one family member; ++, family history of colon, endometrial or ovarian cancer in first degree relative. [b]Previous history of any type of cancers. [c]L, left; R, right colon; NA, not available.

**Figure 1** PCR analysis of instability-prone loci in matched paired normal (N) and tumoral (T) DNA samples from patients affected with sporadic colorectal carcinoma. Four instability prone loci were analysed: 3–381 (**A**), 4–567 (**B**), 4–611 (**C**), 3–896 (**D**). A change in size of microsatellite alleles in tumour cells is considered as instability. Patients assigned as RER+ are denoted by the same number as in Table 2. RER+ patients that do not display a RER phenotype at a particular locus (Table 2) are marked by the number and an asterisk. RER– patients are indicated only by an asterisk reaction was performed by adding 1 U of *Taq* DNA polymerase (BRL) at 80°C, followed by 35 amplification cycles (30 s at 94°C, 45 s at annealing temperature and 30 s at 72°C) and a final extension at 72°C for 7 min. Annealing temperatures were 50°C for loci 4–567 and 3–896 and 58°C and 60°C respectively for loci 3–381 and 4–611. PCR products were analysed on a 6% denaturing polyacrylamide gel. Matched DNA sample pairs were also genotyped using the microsatellite markers DMD, BAT-26, D3S1611, D3S1286, D9S169, D12S320, THRA and D21S1436 as described previously (Baccichet et al. 1997). Information on microsatellites and the primer sequences is available at the Human Genome Database.

### RESULTS AND DISCUSSION

Genetic instability affecting the four loci tested is revealed by the presence of new shorter alleles (Figure 1). These changes were due to the contractions of poly(A) tails of Alu elements (Krajinovic et

© Cancer Research Campaign 1998

**Table 2** Extended microsatellite analysis of tumours displaying instability in at least one locus

| Patient | Cancer site | 3–381[a] | 4–567[a] | 4–611[a] | 3–896[a] | DMD | BAT-26 | D3S1611 | D3S1286 | D9S169 | D12S320 | THRA | D21S1436 |
|---|---|---|---|---|---|---|---|---|---|---|---|---|---|
| 1 | Colon | + | + | + | + | + | + | – | + | +[b] | – | +[b] | + |
| 2 | Colon | – | +[b] | + | + | LOH | +[b] | – | +[b] | + | + | LOH | + |
| 3 | Colon | – | + | + | + | + | + | +[b] | + | – | +[b] | – | – |
| 4 | Colon | + | + | + | + | + | + | LOH | – | +[b] | + | + | + |
| 5 | Colon | + | + | + | – | – | + | – | – | – | – | – | – |
| 6[c] | Colon | – | – | +[b] | – | – | – | LOH | LOH | – | – | – | – |
| 7 | Colon | – | +[b] | – | – | – | – | – | – | – | – | +[b] | – |
| 8 | Endometrium | + | + | + | + | + | + | – | + | – | + | + | + |
| 9 | Endometrium | + | + | + | + | + | + | – | – | + | LOH | – | – |
| 10 | Endometrium | + | + | + | + | ND | ND | ND | ND | ND | ND | ND | ND |
| 11 | Endometrium | – | – | +[b] | – | – | – | – | – | – | – | – | + |
| 12[c] | Ovary | – | – | + | – | – | – | – | – | – | – | LOH | – |
| 13 | Ovary | + | + | + | + | + | + | + | – | +[b] | + | + | LOH |
| 14 | Ovary | – | – | +[b] | – | – | + | + | + | LOH | + | – | – |

[a]Loci susceptible to replication error described in this study. [b]Microsatellite instability type II (difference equal to or less than two repeat units). the other unstable loci show a type I alteration (additional allele is different by more than two repeat units). [c]Patients with microsatellite instability at only one locus. ND. Not done: + denotes the presence of microsatellite instability: LOH. loss of heterozygosity. although the finding that appears as LOH could not be distinguished with certainty from instability in the cases of polymorphic markers.

**Figure 2** Sensitivity of microsatellite markers to detect RER. Fraction of the RER+ tumours identified by a given marker is indicated on the y-axis (per cent)

al. 1996). Tumours were classified as RER+ when additional alleles were observed in more than one marker locus in tumour cells when compared with the allelic pattern obtained from the normal cells of the same patient (Figure 1. Table 2). Replication errors were detected in at least one of our markers in 7 out of 25 of the sporadic colorectal carcinoma cases. Six (24%) of these patients displayed a RER+ phenotype (Table 2). Therefore. the four loci were efficient in the detection of a RER+ phenotype in the sporadic form of colorectal cancers. This frequency falls within the range of 12–28% reported in the literature (Ionov et al. 1993: Thibodeau et al. 1993: Kim et al. 1994). However. no instability at the same loci was detected in breast (22 cases) or in lung (27 cases) cancers (data not shown). which is not concordant with literature data (Eschelman and Markowitz. 1995) reporting that up to 45% of lung and 20% of breast carcinomas are of the RER phenotype. This discrepancy could be ascribed to a less stringent definition of the RER phenotype or to differences among the sets of marker loci assayed. For instance. some loci might be more or less sensitive to replication errors. or they could be located within genomic domains sensitive to RER in a tumour-specific manner.

To determine whether the four new markers might also be altered in non-colonic tissue(s) that are usually transformed in HNPCC patients (Eschelman and Markowitz. 1995: Lynch and Smyrk. 1996). we analysed patients affected by endometrial ($n$ = 12) and ovarian ($n$ = 12) cancer. Four (33%) endometrial and three (25%) ovarian carcinoma patients displayed RERs in at least one of the new loci analysed. although only two (2/12. 17%) of the ovarian carcinomas were considered RER+ (Table 2). The occurrence of the RER phenotype in endometrial carcinoma was higher than reported previously (Eschelman and Markowitz. 1995). The sensitivity of 3–381. 4–567. 4–611 and 3–896 to reveal RERs was compared with eight other microsatellites. both mono- (BAT-26 and DMD loci) and dinucleotide repeats. that were used by other groups to study microsatellite instability. The results for cases only in which microsatellite instability was found are shown in Table 2 and Figure 2. Loci 4–567 and 4–611 together with BAT-26 appeared preferentially to display the RER phenotype in the analysed carcinomas (Figure 2). Marker 4–611 was shown to be as good as BAT-26 (Table 2) in detecting highly unstable cases (10/10). although its predictive value seems to be limited because of false-positive cases (patients 6 and 12. Table 2). The other markers (3–381. 4–567 and 3–896) were less sensitive. detecting respectively seven. nine and eight of the ten highly unstable cases. However. markers 3–381 and 3–896 showed greater susceptibility to RER than any dinucleotide and the DMD mononucleotide repeats tested (Figure 2). Interestingly. in one of the RER+ tumours (patient 5). instability was seen at four markers with mononucleotide repeats (including BAT-26) but not at a single dinucleotide repeat. thus reinforcing the notion that the former markers have a good predictive value for RER. These results indicate that the four new marker loci. particularly 4–567 and 4–611. are sensitive to RER in sporadic carcinomas affecting the same tissues as in cancer patients from HNPCC families.

Table 1 shows that the RER phenotype tends to be more frequent in colorectal carcinoma patients with a familial history of cancer. proximal location. the presence of polyps and the absence of lymph node involvement. i.e. with the clinical features known to be associated with HNPCC-related colon cancers (Kim et al. 1994: Liu et al. 1995$a$. 1995$b$: Moslein et al. 1996: Muta et al. 1996). The

© Cancer Research Campaign 1998 

Table 3 Polymorphic status of 3–381, 4–567, 4–611 and 3–896 mononucleotide repeats

| Locus name | Chromosomal localization[a] | Number of individuals analysed | Allele number/ allele range (%)[b] |
|---|---|---|---|
| 3–381 | 7q22.3 | 46 | 3/130–132 bp (94%) |
| 4–567 | 6p25.1 | 51 | 7/97–105 bp[c] |
| 4–611 | 2q35 | 61 | 4/68–73 bp (78%) |
| 3–896 | 16q23.2 | 46 | 1/240 bp (100%) |

[a]Krajinovic et al (1997). [b]Frequency of the most common allele for loci 3–381, 4–611 and 3–896 is given in parenthesis. [c]Highly polymorphic marker.

pattern of RER phenotype was analysed with respect to the number of loci altered and the degree to which the allele in tumour tissue was shifted (Table 2). A pattern similar to that seen in colorectal carcinoma was also observed in endometrial and ovarian cancers. Most patients showed alterations at multiple loci with pronounced differences between shifted and normal alleles. This, so-called type I alteration (in which the additional allele is shifted by more than two repeat units) is a feature known to be typical for RER+ HNPCC cells (Thibodeau et al, 1993). The fact that microsatellite loci particularly sensitive to RER in cancer cells display similar alterations in sporadic cases suggests similarities in the deficiency of the repair system. On the other hand, patient 7, who had colorectal carcinoma, and patient 11, who had endometrial carcinoma (Table 2), displayed a weaker RER phenotype (two loci altered) that is associated with type II alterations, which shift in size by only one or two repeat units (Table 2). Owing to the small number of patients with this phenotype, it is not possible to establish the predictive value of our markers in such cases. However, this finding might reflect different gene defects or alterations in distinct mismatch repair pathways, as suggested by different mutational spectra in DNA mismatch repair genes in sporadic colon cancers and HNPCC (Liu et al, 1995*a*; Moslein et al, 1996).

Here, we demonstrated that the alteration of loci sensitive to RER in the sporadic HNPCC-related cancers is similar to one observed in hereditary forms of these tumours. The fact that HNPCC patients are prone to develop particular tumours led to the suggestion that specific genomic domains might constitute preferential targets of RER in a tissue-dependent manner. Several documented examples (Mao et al, 1994; Shibata et al, 1994; Parsons et al, 1995; Krajinovic et al, 1996; Malkhosyan et al, 1996; Hoang et al, 1997; Rampino et al, 1997) suggested that locus sensitivity to RERs could be related to certain characteristics, such as the sequence context, the chromatin organization or the expression and replication status of chromosomal domains (review in Coleman and Tsongalis, 1995). For instance, we and others have noticed that loci susceptible to displaying a RER phenotype are not necessarily polymorphic (Jarnik et al, 1996; Krajinovic et al, 1996; Hoang et al, 1997). The loci 3–381 and 4–611 are barely polymorphic in the population, whereas at the locus 3–896 no variability was observed among normal individuals (Table 3). The apparent tissue specificity may also reflect an increased susceptibility of the RER+ cells to exposure to specific mutagens at the colorectal level. It has recently been proposed that mismatch repair genes might play a significant role in carcinogenesis by fixing the environmentally induced DNA lesions (Holzman, 1996). The understanding of how certain chromosomal regions are more susceptible than others to the expression of a RER phenotype opens the possibility of improving this diagnostic tool for certain cancer types, more specifically for those in which prognosis value is associated with the RER status.

### ACKNOWLEDGEMENTS

We are indebted to Dr Lj Lukovic (Institute of Biology and Human Genetics, Belgrade) Drs J Lazic and N Ostojic (Clinic and Institute of Gynaecology and Obstetrics, Belgrade) for providing some DNA samples, and R Ballarano for typing the manuscript. This research was supported by the Fondation de l'Hôpital Sainte-Justine and the Fondation Charles Bruneau. DS is a scholar of the Fonds de recherche en santé du Québec.

### REFERENCES

Baccichet A, Benachenhou N, Couture F, Leclerc JM and Sinnett D (1997) Microsatellite instability in childhood T-cell acute lymphoblastic leukemia. *Leukemia* **11**: 797–802

Coleman WB and Tsongalis GJ (1995) Multiple mechanisms account for genomic instability and molecular mutation in neopastic transformation. *Clin Chem* **41**: 644–657

de la Chapelle A and Peltomaki P (1995) Genetics of hereditary colon cancer. *Annu Rev Genet* **29**: 329–348

Eschelman JR and Markowitz SD (1995) Microsatellite instability in inherited and sporadic neoplasms. *Curr Opinion Oncol* **7**: 83–89

Hoang J-M, Cottu PH, Thuille B, Salmon RJ, Thomas G and Hamelin R (1997) BAT-26, an indicator of the replication error phenotype in colorectal cancers and cell lines. *Cancer Res* **57**: 300–303

Holzman D (1996) Mismatch repair genes matched to several new roles in cancer. *J Natl Cancer Inst* **88**: 950–951

Ionov Y, Peinado MA, Malkhosyan S, Shibata D and Perucho M (1993) Ubiquitous somatic mutations in simple repeated sequences reveal a new mechanism for colonic carcinogenesis. *Nature* **363**: 558–561

Jarnik M, Tang JQ, Korab-Laskowska M, Zietkiewicz E, Cardinal G, Gorska-Flipot I, Sinnett D and Labuda D (1996) Overall informativity, OI, in DNA polymorphisms revealed by inter-Alu PCR. Detection of genomic rearrangements. *Genomics* **36**: 388–398

Kane MF, Loda M, Gaida GM, Lipman J, Mishra R, Goldman H, Jessup JM and Kolodner R (1997) Methylation of the hMLH1 promoter correlates with lack of expression of hMLH1. *Cancer Res* **57**: 808–811

Kim H, Jen J, Vogelstein B and Hamilton SR (1994) Clinical and pathological characteristics of sporadic colorectal carcinomas with DNA replication errors in microsatellite sequences. *Am J Pathol* **145**: 148–156

Kolodner RD (1995) Mismatch repair: mechanisms and relationship to cancer susceptibility. *Trends Biol Sci* **20**: 397–401

Krajinovic M, Richer C, Labuda D and Sinnett D (1996) Detection of a RER+ phenotype in cancer cells by inter-Alu polymerase chain reaction. *Cancer Res* **56**: 2733–2737

Krajinovic M, Richer C, Lukovic L, Labuda D and Sinnett D (1997) Chromosomal assignment of loci susceptible to replication errors by radiation hybrid mapping. *Mutat Res Genomics* **382**: 81–83

Liu B, Nicolaides NC, Markowitz S, Willson JKV, Parsons RE, Jen J, Papadopolous N, Peltomaki P, de la Chapelle A, Hamilton SR, Kinzler KW and Wogelstein B (1995*a*) Mismatch repair gene defects in sporadic colorectal cancers with microsatellite instability. *Nature Genet* **9**: 48–55

Liu B, Farrington SM, Petersen GM, Hamilton SR, Parsons R, Papadopoulus N, Fujiwara T, Jen J, Kinzler KW, Wyllie AH, Vogelstein B and Dunlop MG (1995*b*) Genetic instability occurs in the majority of young patients with colorectal cancers. *Nature Med* **1**: 348–352

Lynch HT and Smyrk T (1996) Hereditary non-polyposis colorectal cancer (Lynch Syndrome). *Cancer* **78**: 1149–1167

Malkhosyan S, Rampino N, Yamamoto H and Perucho M (1996) Frameshift RER+ mutations. *Nature* **382**: 499–500

Mao L, Lee DJ, Tockman MS, Erozan YS, Askin F, Sidransky D (1994) Microsatellite alterations as clonal markers for the detection of human cancer. *Proc Natl Acad Sci USA* **91**: 9871–9875

Moslein G, Tester DJ, Lindor NM, Honchel R, Cunningham JM, French AJ, Halling KC, Schwab M, Goretzki P and Thibodeau SN (1996) Microsatellite instability and mutation analysis of hMSH2 and hMLH1 in patients with sporadic, familial and hereditary colorectal cancer. *Hum Mol Genet* **9**: 1245–1252

© Cancer Research Campaign 1998

Muta H. Noguchi M. Perucho M. Ushio K. Sugihara K. Ochiai A. Nawata H and Hirohashi S (1996) Clinical implications of microsatellite instability in colorectal cancer. *Cancer* **77**: 265–270

Parsons R. Myeroff LL. Liu B. Willson JKV. Markowitz SD. Kinzler KW and Vogelstein B (1995) Microsatellite instability and mutations of the transforming growth factor type II receptor gene in colorectal cancer. *Cancer Res* **55**: 5548–5550

Rampino N. Yamamoto H. Ionov Y. Li Y. Sawai H. Reed JC and Perucho M (1997) Somatic frameshift mutations in the *BAX* gene in colon cancers of the microsatellite mutator phenotype. *Science* **275**: 967–969

Shibata D. Peinado MA. Ionov Y. Malkhosyan S and Perucho M (1994) Genomic instability in repeated sequences is an early somatic event in colorectal tumorigenesis that persists after transformation. *Nature Genet* **6**: 273–280

Thibodeau SN. Bren G and Schaid D (1993) Microsatellite instability in cancer of the proximal colon. *Science* **260**: 816–819

Thibodeau SN. French AJ. Roche PC. Cunningham JM. Tester DJ. Lindor NM. Moslein G. Baker SM. Liskay RM. Burgart LJ. Honchel R and Halling KC (1996) Altered expression of hMSH2 and hMLH1 in tumors with microsatellite instability and genetic alterations in mismatch repair genes. *Cancer Res* **56**: 4836–4840

© Cancer Research Campaign 1998